# Prostate Cancer Mortality in Men Aged 70 Years Who Recently Underwent Prostate-Specific Antigen Screening

**DOI:** 10.1001/jamanetworkopen.2024.59766

**Published:** 2025-02-14

**Authors:** Dana H. Chung, Tanner J. Caverly, Matthew J. Schipper, Timothy P. Hofer, Roman Gulati, Brent S. Rose, Megan E. V. Caram, Phoebe A. Tsao, Kristian D. Stensland, David Elliott, Sameer D. Saini, Alex K. Bryant

**Affiliations:** 1 University of Michigan Medical School, Ann Arbor; 2 Veterans Affairs Center for Clinical Management Research, Ann Arbor, Michigan; 3 Department of Internal Medicine, University of Michigan, Ann Arbor; 4 Division of Hematology/Oncology, Department of Internal Medicine, University of Michigan, Ann Arbor; 5 Department of Urology, University of Michigan, Ann Arbor; 6 Department of Radiation Oncology, Veterans Affairs Ann Arbor Healthcare System, Ann Arbor, Michigan; 7 Department of Radiation Oncology, University of Michigan, Ann Arbor; 8 Department of Biostatistics, University of Michigan, Ann Arbor; 9 Division of Public Health Sciences, Fred Hutchinson Cancer Center, Seattle, Washington; 10 Department of Radiation Medicine and Applied Sciences, University of California, San Diego, La Jolla

## Abstract

**Importance:**

Continuing prostate-specific antigen (PSA) screening after age 70 years might benefit men at high risk of prostate cancer–specific mortality (PCSM) or metastatic prostate cancer (mPCa), but the relative value of clinical factors (race and ethnicity, competing mortality, and PSA history) in identifying men at higher vs lower risk is unknown.

**Objective:**

To examine the value of PSA levels, race and ethnicity, and competing mortality in risk stratification for PCSM and mPCa in men after age 70 years.

**Design, Setting, and Participants:**

In this cohort study, clinical data of all men receiving health care through the Veterans Health Administration who turned age 70 years between 2008 and 2020 and had a normal screening PSA value between age 65 and 69 years (<4 ng/mL [baseline PSA]) and no prior history of prostate cancer or biopsy were examined. The data cutoff date was December 26, 2023.

**Exposure:**

The most recent screening PSA value from age 65 to 69 years, self-reported race and ethnicity, and competing mortality risk derived from a machine learning model.

**Main Outcome and Measures:**

The 10-year absolute risk of PCSM and mPCa were determined using regression modeling.

**Results:**

The cohort included 921 609 men who turned 70 years between 2008 and 2020; 11% of whom self-reported as Black and 82% as White race. Between age 65 and 70 years, 45% of patients had a baseline PSA of less than 1.00 ng/mL, and 32% had a baseline PSA of 1.00 to 1.99 ng/mL. Most patients (87%) continued to undergo screening past age 70 years, with little variation by competing mortality risk or race and ethnicity. The 10-year cumulative incidence of PCSM was 0.26% overall, and 95% of men had a 10-year risk less than 0.73%. Higher baseline PSA level between age 65 and 69 years was associated with 10-year PCSM risk (0.79% for 3.00-3.99 ng/mL vs 0.10% for 0.20-0.99 ng/mL), race and ethnicity (0.36% for Black vs 0.25% for White), and competing mortality (0.24% for the highest quintile vs 0.21% for the lowest quintile). Similar results were found for mPCa. Low PSA (0.20-0.99 ng/mL) was associated with very low PCSM and mPCa risk, even among Black men in the healthiest quintile of competing mortality risk (10-year PCSM risk, 0.08% [95% CI, 0.01%-0.44%]; 10-year mPCa risk 0.24% [95% CI, 0.10%-0.52%]).

**Conclusions and Relevance:**

In this cohort study, the findings suggest that most men receiving care through the VHA continue PSA screening after age 70 years despite low absolute 10-year PCSM risks. The PSA values from age 65 to 69 years may be highly informative for adverse prostate cancer outcomes after age 70 years, with a PSA less than 1 ng/mL associated with a very low risk of long-term PCSM and mPCa.

## Key Points

**Question:**

What are the associations among race and ethnicity, competing mortality risk, and prostate-specific antigen (PSA) history with respect to prostate cancer–specific mortality (PCSM) and metastatic prostate cancer (mPCa) among screened men after age 70 years?

**Findings:**

In this cohort study of 921 609 men, PSA values from age 65 to 69 years were associated with 10-year PCSM and mPCa, regardless of race and ethnicity or competing mortality. Low PSA (<1 ng/mL) was associated with very low PCSM and mPCa risk, including among the healthiest Black men.

**Meaning:**

These findings suggest that PSA values before age 70 years may be highly informative for long-term PCSM and mPCa risk, with a PSA of less than 1 ng/mL identifying a very-low-risk subgroup.

## Teaser

This cohort study examines prostate cancer–specific mortality and metastatic prostate cancer risks after age 70 in men who had undergone recent prostate-specific antigen (PSA) screening.

## Introduction

The potential benefit of prostate-specific antigen (PSA) screening in older men (aged ≥70 years) is controversial. While the risk of prostate cancer–specific mortality (PCSM) increases dramatically with age, competing mortality risks may erode any net benefits from screening. Thus, uncertainty exists about the benefit of continuing screening past age 70 years and the applicability of randomized evidence for PSA screening in older men with multiple comorbidities.^1,2,3^ As a result, practice guidelines vary in the recommended upper age limit of screening from 70 years^4^ to more than 75 years, depending on general health, prior PSA values, and patient preferences after shared decision-making.^5,6^ Black men are known to be at substantially higher risk of PCSM compared with other racial and ethnic groups, and it is possible that Black men might preferentially benefit from continued screening past age 70 years, though randomized data are lacking to support this conjecture.^7^ Despite these ambiguities, continued screening after age 70 years remains widespread in the US^8^ and drives high rates of overdiagnosis.^9,10^

Primary care physicians must consider a number of clinical factors when deciding whether to continue screening older men, including prior PSA screening history, family history, race and ethnicity, and competing mortality risk. Current guidelines differ in their relative emphasis on each factor,^4,5^ and physicians lack clear guidance on which factors should be emphasized during shared decision-making. In this study, we leverage a large, diverse, national cohort of previously screened men as they turned 70 years to examine the value of PSA values, race and ethnicity, and competing mortality risk in identifying those at elevated risk of PCSM and metastatic prostate cancer (mPCa) who might preferentially benefit from continued screening. We hypothesized that prior screening PSA values may be highly informative (in combination with self-reported race and ethnicity) for the risk of PCSM and mPCa after age 70 years.

## Methods

### Data Source

This cohort study used electronic medical record (EMR) data from the Veterans Health Administration (VHA) Corporate Data Warehouse, which includes EMR data for all veterans receiving care through VHA facilities nationwide. To capture procedures and diagnoses outside of VHA, we included linked Medicare claims data and VHA community care data covering non-VHA care. This study was approved by the Veterans Affairs Ann Arbor Institutional Review Board. Health Insurance Portability and Accountability Act authorization was waived because the study analyzed retrospective data and involved minimal risk to participants. This study followed the Strengthening the Reporting of Observational Studies in Epidemiology (STROBE) reporting guideline.

### Cohort Definition

We included all male veterans who turned age 70 years between 2008 and 2020 and had at least 1 PSA screen between age 65 and 69 years (eFigure 1 in Supplement 1). The index date was defined as the first VHA encounter during the year they were aged 70 years. We excluded patients whose PSA was less than 0.20 ng/mL (to exclude those who may have been previously treated for prostate cancer) or greater than 4.00 ng/mL (to exclude those for whom prostate biopsy would be potentially indicated) (to convert to micrograms per liter, multiply by 1). For patients with positive results for 5-α reductase inhibitors (5-ARIs), the most recent PSA value before age 70 years was corrected by multiplying by 2.^11^ We excluded patients who had less than 5 years of clinical data before the index date and patients with a previous diagnosis of prostate cancer (by *International Classification of Diseases, Ninth Revision* and *International Statistical Classification of Diseases, Tenth Revision* [*ICD-9*/*10*] code) or prostate biopsy (by *Current Procedural Terminology* [*CPT*] and *ICD-9*/*10* Procedure Coding System code). We further excluded the less than 5% of patients with missing or inconsistent covariate or follow-up information. In total, 35% of the cohort was excluded.

### PSA Screening During Follow-Up and Prostate Cancer Outcomes

We identified PSA screens during follow-up through structured VHA laboratory data, excluding any PSA tests that occurred after a prostate cancer diagnosis. Prostate biopsies were identified through *CPT* and *ICD-9*/*10* Procedure Coding System codes. Metastatic prostate cancer diagnoses were identified through a validated natural language processing algorithm that identifies new mentions of mPCa in clinical notes and radiographic reports.^12,13^ Prostate cancer–specific mortality was identified through the underlying cause of death in linked National Death Index death certificate data. As National Death Index data were only available through 2021, we truncated follow-up on December 31, 2021, for all PCSM analyses. Date of death was obtained from the Death Ascertainment File, a VHA database that collects death information from multiple governmental sources. We also ascertained rates of diagnosis of clinically significant prostate cancer, defined as any prostate cancer of Gleason 3 + 4 or higher, ascertained through a validated natural language processing algorithm.^12^ Finally, we ascertained prostate cancer treatment (radiotherapy within 90 days after a prostate cancer diagnosis, prostatectomy, or hormonal therapy on the basis of *CPT* codes, *ICD-9*/*10* procedure codes, and pharmacy records) as an additional outcome.

### Baseline Covariates

We defined baseline PSA as the most recent PSA screen between age 65 and 69 years (ie, within 5 years before the index date when the patient turned 70 years). Baseline PSA was stratified into 4 levels (0.20-0.99, 1.00-1.99, 2.00-2.99, and 3.00-3.99 ng/mL). Other baseline characteristics taken from the EMR included self-reported race and ethnicity (Hispanic, non-Hispanic Black [henceforth, Black], non-Hispanic White [henceforth, White], or other); the number of PSA screens in the 5 years prior to the index date; the presence of primary care, emergency department, or urologist encounters in the prior year; use of 5-ARIs, α-1 antagonists, or phosphodiesterase-5 inhibitors in the prior year; baseline diagnosis of benign prostatic hypertrophy or prostatitis in the prior year (by *ICD 9*/*10* codes); geographic region; Charlson Comorbidity Index^14^; and VA Frailty Index.^15^ Baseline PSA screening was defined as the most recent PSA value for each patient who turned 65 to 69 years from 2008 to 2020. Recognizing that racial and ethnic disparities in prostate cancer outcomes may reflect an interconnected combination of social, environmental, and genetic influences, we use self-reported race and ethnicity in this study as a proxy for these complex and intersecting factors. Other races included American Indian or Alaska Native, Asian, and Native Hawaiian or Pacific Islander. To capture competing mortality risk, we developed a machine learning model using structured EMR data to stratify patients with respect to their 10-year all-cause mortality risk. Details of the model development and estimating variables are presented in the eMethods and eTable 1 in Supplement 1.

### Statistical Analysis

We estimated the yearly postbaseline PSA screening rate by dividing the number of patients with a PSA screen during each year of follow-up by the number of patients alive and free of prostate cancer each year. The mean cumulative number of PSA screens over time was estimated using the unadjusted mean cumulative count, accounting for the competing risk of death and censoring.^16^ We censored patients at the time of death, prostate cancer diagnosis, or last follow-up, whichever occurred first. To assess the association between baseline patient characteristics and the intensity of PSA screening during follow-up, we performed multivariable negative binomial regression using an offset term equal to the natural log of follow-up time. We estimated absolute rates of prostate biopsies and prostate cancer diagnoses using the cumulative incidence method accounting for the competing risk of death. Patients were followed up until the date of death or last follow-up in the VHA system. The data cutoff date was December 26, 2023.

We developed an absolute risk regression model^17^ to determine 10-year risk of PCSM accounting for competing all-cause mortality. Variables included in this model were self-reported race and ethnicity, estimated 10-year survival probability decile from the survival estimation model, and baseline PSA. Baseline PSA was modeled as a natural spline with 3 *df* to account for a nonlinear association with PCSM risk. Calibration of the integrated model was assessed using plots of observed vs estimated 10-year PCSM risk by decile of estimated risk (eFigure 2 in Supplement 1). Observed risk in each decile was calculated using the cumulative incidence method. To account for potential overfitting, patient-level 10-year PCSM risk estimations from each model were generated through 10-fold cross validation, where each patient’s estimate was derived from a risk model that did not include the patient’s data in the model fitting process.^18^ In additional analyses, we repeated this modeling process with mPCa as the primary outcome and within subgroups defined by self-reported race and ethnicity and in the highest and lowest estimated survival quintiles. Metastatic prostate cancer was used as the outcome in these subgroup analyses because of low PCSM event rates within racial and ethnic and estimated survival subgroups.

 The statistical analysis was performed using R, version 4.3.1 (R Foundation) and Python, version 3.11.4 (Python Software Foundation). The threshold for significance is a 2-sided *P* < .05.

## Results

### Cohort Characteristics

After exclusions, the cohort included 921 609 patients who turned 70 years between 2008 and 2020, of whom 11% were of Black, 5% Hispanic, 82% White, and 2% other race and ethnicity (Table). We found that 45% of patients had a baseline PSA of between 0.20 and 0.99 ng/mL, 32% had between 1.00 and 1.99 ng/mL, 15% had between 2.00 and 2.99 ng/mL, and 8% had between 3.00 and 3.99 ng/mL. Patients in the higher baseline PSA groups were more likely to be taking 5-ARIs and α-1 antagonist medications at baseline, to have seen a urologist in the prior year, and to have a history of benign prostatic hypertrophy. Baseline patient characteristics by racial and ethnic group, estimated 10-year survival quintile, and estimated 10-year PCSM risk quintile are shown in eTables 2 through 4 in Supplement 1. The overall survival model showed good calibration (eFigure 2 in Supplement 1) and discrimination (C index, 0.75; Brier score, 0.15 on held out test set).

**Table.** Sample Characteristics by Baseline PSA (N = 921 609)^a^

Characteristic	Patients, No. (%)			
	PSA 0.20-0.99 ng/mL	PSA 1.00-1.99 ng/mL	PSA 2.00-2.99, ng/mL	PSA 3.00-3.99 ng/mL
No. of patients	415 444 (45)	296 490 (32)	137 274 (15)	72 401 (8)
Self-reported race and ethnicity				
Hispanic	19 783 (5)	14 573 (5)	6717 (5)	3646 (5)
Non-Hispanic Black	42 858 (10)	33 619 (11)	16 809 (12)	9406 (13)
Non-Hispanic White	343 362 (83)	241 619 (81)	110 636 (81)	57 727 (80)
Other^b^	9441 (2)	6679 (2)	3112 (2)	1622 (2)
Most recent PSA, median (IQR), ng/dL	0.60 (0.40-0.78)	1.39 (1.17-1.64)	2.40 (2.19-2.67)	3.40 (3.20-3.68)
No. of PSA screens in prior 5 y, median (IQR)	4 (3-5)	4 (3-5)	4 (3-6)	5 (3-6)
Index date, y				
2008-2010	44 441 (11)	31 759 (11)	14 496 (11)	7402 (10)
2011-2013	66 215 (16)	47 040 (16)	21 496 (16)	11 027 (15)
2014-2016	109 519 (26)	78 114 (26)	36 406 (27)	19 257 (27)
2017-2020	195 269 (47)	139 577 (47)	64 876 (47)	34 715 (48)
Geographic region				
Continental	73 654 (18)	51 238 (17)	23 365 (17)	12 426 (17)
Midwest	100 263 (24)	71 766 (24)	33 251 (24)	17 291 (24)
North Atlantic	93 641 (23)	66 719 (23)	30 955 (23)	16 233 (22)
Pacific	63 314 (15)	46 373 (16)	21 734 (16)	11 861 (16)
Southeast	84 572 (20)	60 394 (20)	27 969 (20)	14 590 (20)
Area deprivation index				
1-20 (Least disadvantage)	35 250 (9)	26 358 (9)	12 345 (9)	6801 (10)
21-40	67 316 (17)	49 239 (17)	23 239 (17)	12 258 (17)
41-60	93 363 (23)	67 600 (23)	31 001 (23)	16 291 (23)
61-80	108 100 (27)	75 444 (26)	34 932 (26)	18 159 (26)
81-100 (Highest disadvantage)	103 647 (25)	72 233 (25)	33 151 (25)	17 561 (25)
RUCA classification				
Urban	302 223 (73)	216 688 (73)	100 528 (74)	53 132 (74)
Large rural city or town	56 443 (14)	39 813 (13)	18 036 (13)	9542 (13)
Small rural town	30 831 (8)	21 319 (7)	10 047 (7)	5181 (7)
Isolated small rural town	24 326 (6)	17 558 (6)	8106 (6)	4258 (6)
Distance from VHA facility, miles				
0-25	264 574 (65)	190 990 (65)	89 056 (66)	46 990 (66)
26-50	77 443 (19)	54 051 (19)	24 791 (18)	12 872 (18)
51-75	32 589 (8)	22 671 (8)	10 313 (8)	5544 (8)
>75	34 158 (8)	24 092 (8)	10 926 (8)	5857 (8)
Charlson Comorbidity Index, median (IQR)^c^	1 (0-3)	1 (0-3)	1 (0-3)	1 (0-3)
VA Frailty Index, median (IQR)^d^	0.16 (0.10-0.26)	0.16 (0.10-0.26)	0.16 (0.10-0.23)	0.13 (0.10-0.23)
History of BPH	131 478 (32)	103 251 (35)	55 100 (40)	32 763 (45)
History of prostatitis	14 203 (3)	11 699 (4)	6201 (5)	4027 (6)
5-ARI use at baseline	22 429 (5)	18 343 (6)	11 475 (8)	7988 (11)
α-1 Antagonist use at baseline	81 776 (20)	64 207 (22)	34 952 (25)	20 655 (29)
PDE-5 inhibitor use at baseline	77 085 (19)	59 958 (20)	28 559 (21)	15 208 (21)
ED visit in prior year	67 218 (16)	45 459 (15)	20 882 (15)	11 251 (16)
PCP visit in prior year	363 563 (88)	256 951 (87)	118 720 (86)	62 843 (87)
Urologist visit in prior year	26 841 (7)	20 800 (7)	11 615 (9)	8019 (11)

Abbreviations: 5-ARI, 5-α reductase inhibitor; BPH, benign prostatic hypertrophy; ED, emergency department; PCP, primary care physician; PDE-5, phosphodiesterase-5; PSA, prostate-specific antigen; RUCA, Rural-Urban Commuting Area; VA, Veterans Affairs; VHA, Veterans Health Administration.

^a^ Percentages may not add to 100 due to rounding. To convert PSA values to micrograms per liter, multiply by 1.

^b^ Other includes American Indian or Alaska Native, Asian, and Native Hawaiian or Pacific Islander.

^c^ Scale of 0 to 24, with higher numbers indicating more severe comorbidity.

^d^ A score of less than or equal to 0.10 indicates nonfrail; 0.11 to 0.20, prefrail; 0.21 to 0.30, moderately frail; and greater than 0.40, severely frail.

### PSA Screening Patterns

**Figure 1.**
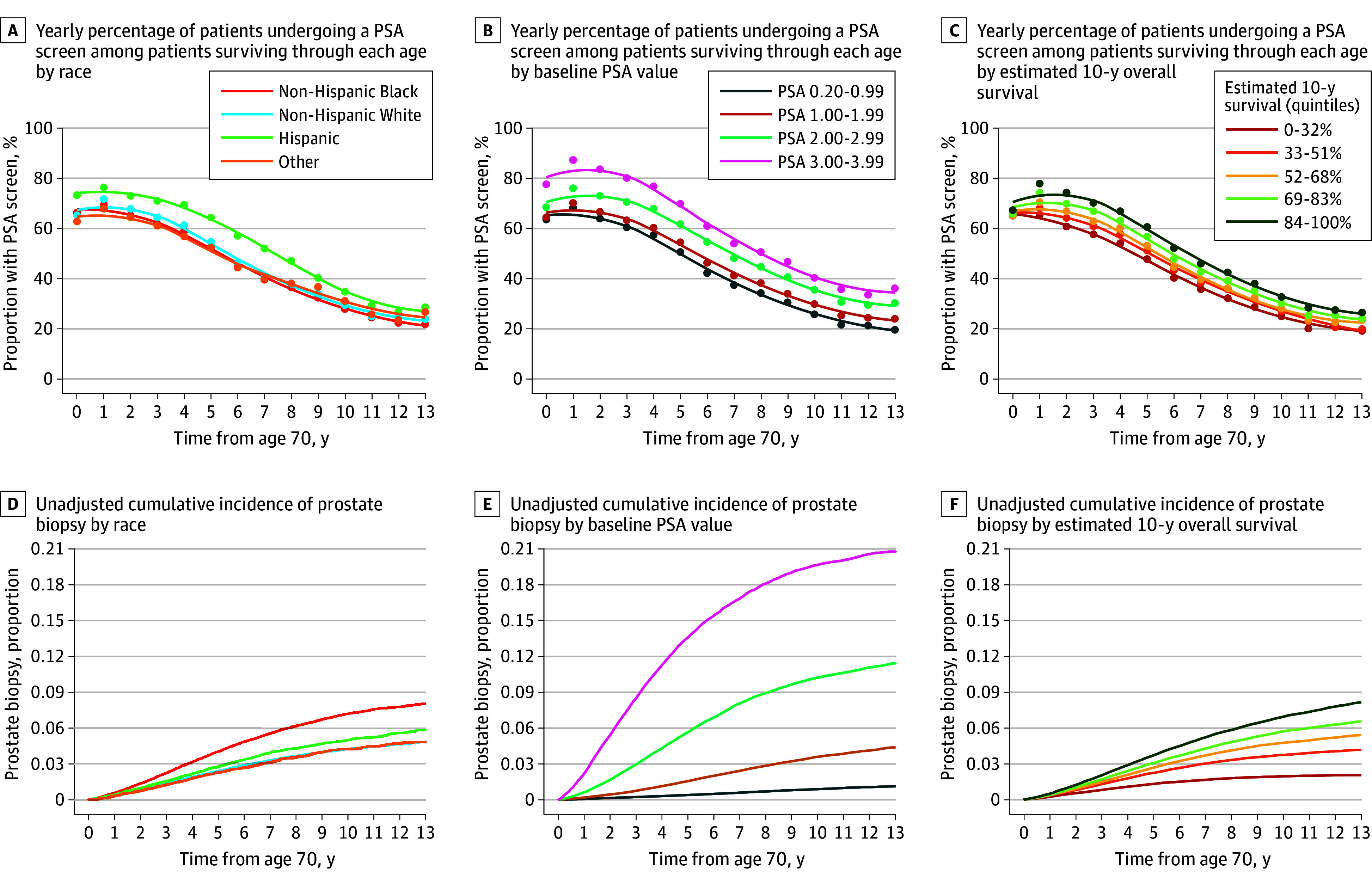
Prostate-Specific Antigen (PSA) Screening and Prostate Biopsy Rates Baseline PSA is the most recent screening PSA between age 65 and 69 years. PSA is measured in nanograms per deciliter; to convert to micrograms per liter, multiply by 1.

Continued PSA screening after age 70 years was almost universal; the cumulative incidence of at least 1 additional screen was 87% by age 80. Screening was widespread in all patient subgroups, including those with low baseline PSA levels and poor estimated 10-year survival (Figure 1A-C). Continued screening among Hispanic patients was slightly more common compared with the other racial and ethnic groups (Figure 1A). A random sample of patient screening histories is shown in eFigure 3 in Supplement 1. While the frequency of yearly screening decreased with advancing age, the yearly screening rate was still approximately 30% among patients who survived to age 80 or older. The mean cumulative number of PSA screens was 5.43 (95% CI, 5.41-5.45) by 10 years of follow-up. In the multivariable negative binomial model, factors associated with higher intensity of continued PSA screening included higher baseline PSA level (PSA 3.00-3.99 vs 0.20-0.99: incidence rate ratio [IRR], 1.27 [95% CI, 1.26-1.28]), at least 1 primary care physician visit in the prior year (IRR, 1.14; 95% CI, 1.13-1.14), increasing number of routine PSA screens in the prior 5 years (IRR, 1.12 per 1 additional screen; 95% CI, 1.12-1.12), Hispanic ethnicity (IRR, 1.10 [95% CI, 1.09-1.11] vs White), and better 10-year estimated survival (for best vs worst quintile: IRR, 1.07; 95% CI, 1.06-1.07) (eTable 5 in Supplement 1).

### Prostate Cancer Outcomes

The cumulative incidence of prostate biopsy was 4.6% by 10 years in the entire cohort (Figure 1D-F). Patients with a higher baseline PSA level had a dramatically increased risk of undergoing a biopsy (19.7% by 10 years for PSA 3.00-3.99 ng/mL vs 0.89% for PSA 0.20-0.99 ng/mL) (Figure 1E). The cumulative incidence of biopsy was also higher among Black patients (7.2% vs 4.3% for White patients) (Figure 1D) and in patients with better 10-year estimated survival (6.9% in highest quintile vs 1.9% in lowest quintile) (Figure 1F). Hispanic patients had a similar 10-year risk of biopsy (4.9%) compared with White patients. Similar results were found for the cumulative incidence of clinically significant prostate cancer diagnosis and prostate cancer treatment, with a strong dependence on baseline PSA for each (clinically significant prostate cancer: 10-year cumulative incidence, 7.41% for PSA 3.00-3.99 ng/mL vs 0.32% for PSA 0.20-0.99 ng/mL; prostate cancer treatment: 10-year cumulative incidence, 8.25% for PSA 3.00-3.99 ng/mL vs 0.37% for PSA 0.20-0.99 ng/mL) (eFigures 4 and 5 in Supplement 1). All-cause mortality rates differed little by race and ethnicity or baseline PSA, but differed greatly by estimated 10-year survival, as expected (eFigure 6 in Supplement 1).

**Figure 2.**
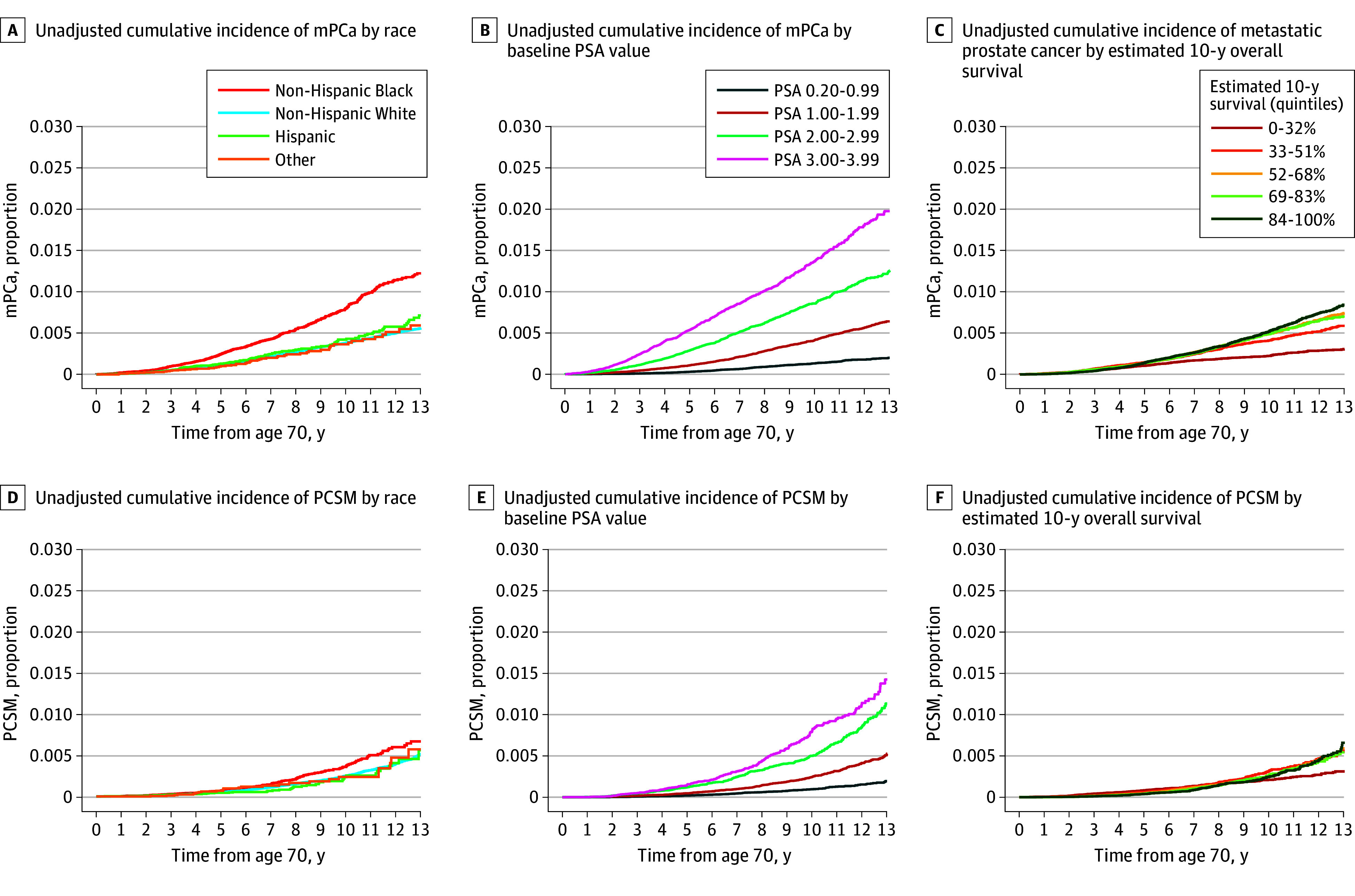
Rates of Adverse Prostate Cancer Outcomes Prostate-specific antigen (PSA) is measured in nanograms per deciliter; to convert to micrograms per liter, multiply by 1. mPCa indicates metastatic prostate cancer; PCSM, prostate cancer–specific mortality.

**Figure 3.**
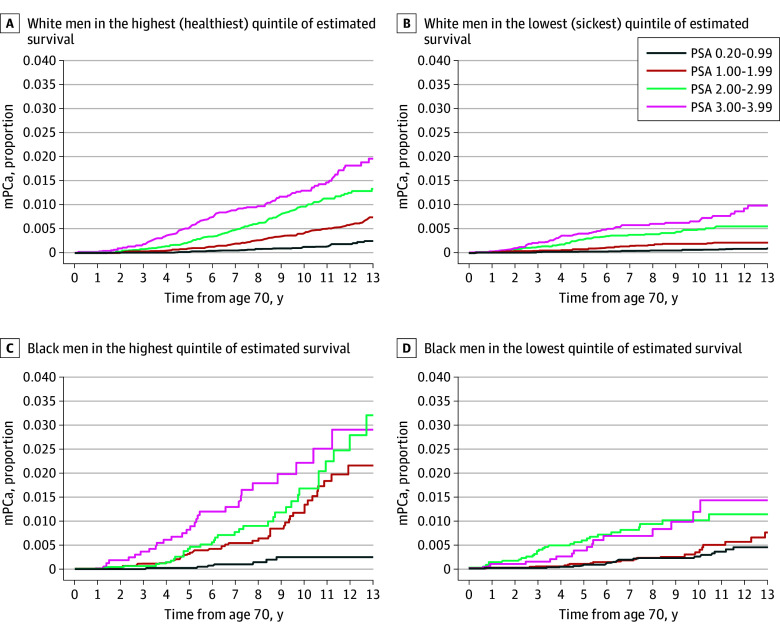
Association of Baseline Prostate-Specific Antigen (PSA) and Metastatic Prostate Cancer (mPCa) Incidence in White and Black Men by Competing Mortality Risk Baseline PSA is the most recent screening PSA between age 65 and 69 years. PSA is measured in nanograms per deciliter; to convert to micrograms per liter, multiply by 1.

The 10-year cumulative incidence of mPCa and PCSM followed similar trends (Figure 2). Higher baseline PSA level was associated with unadjusted 10-year risk of mPCa (1.37% for PSA 3.00-3.99 ng/mL vs 0.13% for PSA 0.20-0.99 ng/mL) and PCSM (0.79% for PSA 3.00-3.99 ng/mL vs 0.10% for PSA 0.20-0.99 ng/mL) (Figure 2B, E). Compared with White patients, Black patients had a higher risk of mPCa (0.79% vs 0.38%) and a somewhat higher risk of PCSM (0.36% vs 0.25%), whereas Hispanic patients had a similar risk (Figure 2A, D). Patients in the poorest estimated survival quintile (highest competing mortality risk) had a somewhat lower absolute risk of mPCa (0.22% vs 0.52%) and PCSM (0.24% vs 0.21%); patients in the remaining quintiles had similar mPCa and PCSM risks (Figure 2C, F). Baseline PSA remained highly prognostic for mPCa in subgroup analyses by race and ethnicity and estimated survival quintile (Figure 3). Even among Black patients in the highest estimated survival quintile, baseline PSA of 0.20-0.99 ng/mL identified patients with a very low 10-year risk of mPCa and PCSM (0.24% [95% CI, 0.10%-0.52%] and 0.08% [95% CI, 0.01%-0.44%], respectively), which remained true in Black patients within the lowest estimated survival quintile and among White patients in the highest or lowest estimated survival quintiles.

### Individual Variation in PCSM Risk

**Figure 4.**
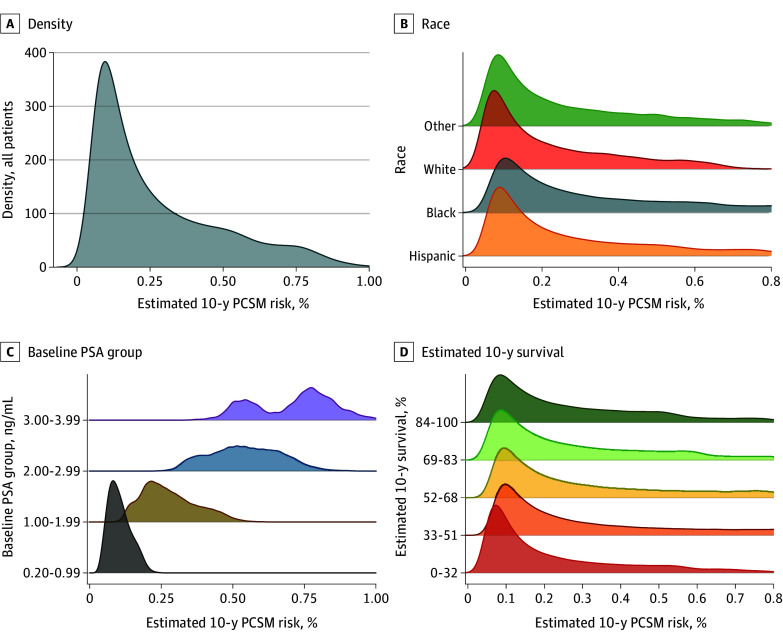
Individual Variation in Estimated Prostate Cancer–Specific Mortality (PCSM) Risk PSA indicates prostate-specific antigen. To convert PSA to micrograms per liter, multiply by 1.

The risk regression model for PCSM showed good calibration (eFigure 2 in Supplement 1). Across the entire cohort, the distribution of estimated 10-year absolute mPCa risk was right-tailed, with a median (IQR) risk of 0.19% (0.10%-0.39%) (Figure 4A). Ninety-five percent of patients had a risk less than 0.73%. Patients with higher baseline PSA had a dramatically higher PCSM risk (PSA 3.00-3.99 ng/mL: median, 0.74% [IQR, 0.57%-0.81%]; PSA 0.20-0.99 ng/mL: median, 0.10% [IQR, 0.08%-0.13%) (Figure 4C). In contrast, risk distributions by race and estimated survival were largely overlapping. Black patients had a slightly higher median PCSM risk (0.24% [IQR, 0.13%-0.50%] vs 0.19% [IQR, 0.10%-0.39%] for White) as did the lowest estimated survival quintile (median, 0.14% [IQR, 0.08%-0.31%] vs 0.19% [IQR, 0.10%-0.39%] for the highest quintile) (Figure 4B, D).

## Discussion

Primary care physicians lack clear guidance on which clinical factors, if any, should guide PSA screening decisions in older men. In this cohort study of more than 900 000 previously screened, racially and ethnically diverse men turning 70 years, we developed individualized risk models to determine long-term PCSM and mPCa using PSA values from age 65 to 69 years, race and ethnicity, and competing mortality risk. We found that PSA has profound prognostic value in differentiating men at higher or lower absolute risk of PCSM and mPCa. In particular, men with a PSA of 0.20-0.99 ng/mL between age 65 and 69 years (which was 45% of our sample) had a very low risk of undergoing prostate biopsy and of mPCa and PCSM, even among the healthiest Black men who would otherwise represent a high-risk subgroup. Meanwhile, men with a PSA of 3.00-3.99 ng/mL, had a substantially elevated risk of PCSM and mPCa compared with men with a lower baseline PSA. These data may inform shared decision-making regarding the potential benefits of continuing PSA screening past age 70 years among healthy men with high-normal PSA values and support the safety of discontinuing screening in men with low PSA values regardless of race and ethnicity or competing mortality risk.

Prior studies have described the influence of PSA values on subsequent risk of deadly prostate cancer. Seminal case-control studies in largely unscreened populations showed that a single PSA in midlife is robustly associated with long-term PCSM risk.^19,20^ Other analyses, such as a recent post hoc analysis of the Rotterdam section of the ERSPC randomized clinical trial, showed that older men with a screening PSA of less than 3 ng/mL, who represent the majority of older screened men, have a less than 1% risk of PCSM and are unlikely to significantly benefit from continued screening.^21^ With some exceptions,^22^ this work has typically focused on men of European ancestry, who are at lower risk of PCSM than men of African ancestry. Furthermore, we lack robust data on how competing mortality interacts with race and ethnicity and PSA to estimate long-term PCSM and mPCa risk. This lack of data creates ambiguity in which clinical factors should carry the most weight in decision making and drives variation across clinical guidelines.^4,5,6^ Given that continued screening among older men remains common (at 87% in our study, even among patients with low baseline PSA levels), clearer guidance is needed.

Our data suggest that a simple assessment of personal risk based on PSA values before age 70 years captures a large proportion of relevant prognostic information with respect to mPCa and PCSM risk. A recent screening PSA of less than 1 ng/mL selects an extraordinarily low-risk population with a less than 1.00% 10-year risk of undergoing prostate biopsy, a diagnosis of clinically significant prostate cancer, and prostate cancer treatment; less than 0.15% risk of mPCa; and less than 0.10% risk of PCSM. This finding remained true even among very healthy Black men, who would otherwise be at higher risk of mPCa and PCSM. While the low rates of prostate biopsies, diagnoses of clinically significant prostate cancer, and prostate cancer treatments in this population suggest a correspondingly low risk of screening-related harm, these men also may not benefit from continued screening. Conversely, men with a higher baseline PSA were at higher risk of mPCa and PCSM, though 10-year PCSM rates were still less than 1%, even among patients in the highest PSA group (3.00-3.99 ng/mL). While healthier men with a high baseline PSA may benefit from continued screening, they are also at higher risk for the harms of prostate biopsy, and many prostate cancers in this population may still be overdiagnosed.^10^ We also found that race and ethnicity had a very limited association with long-term PCSM risk, though slightly larger differences were found in mPCa risk. This finding suggests that differential screening policies by race and ethnicity may have limited benefit among older, previously screened men.^7^ To identify the optimal screening strategies among high-risk older patients, additional modeling studies using emulated randomized clinical trials or natural history models are needed. Our risk estimates may help to inform these efforts.

### Limitations

Our study has several limitations. First, any potential PCSM or mPCa reduction from continued PSA screening past age 70 years is unproven, with a lack of randomized clinical trial data to inform this important question. As such, our results should not be interpreted to support PSA screening among men with higher baseline PSA values; rather, if such a benefit exists, our data suggest that it may be found among men with high baseline PSA. Conversely, our results strongly suggest that men with low baseline PSA levels may not benefit from continued screening because of their very low risk of mPCa and PCSM. Second, continued screening after age 70 years was common in our study population, and our results should be interpreted in this context. If continued screening past age 70 years reduces mPCa and PCSM, rates of these outcomes would be higher in a population with less screening of older men. Third, while we focused on 10-year risks (reflecting risk at age 80 years), a longer time horizon may be increasingly relevant in the future given the increasing life expectancy of recent US birth cohorts. Fourth, while our absolute PCSM rates are similar to previously published estimates from clinical trials,^23^ we relied on death certificates to define PCSM, which may have introduced misclassification error. Fifth, family history of prostate cancer was not included in our analysis because it is not reliably available in EMR data, and we recognize that this may be an additional factor influencing the decision to continue screening past age 70 years. Sixth, prostate cancer treatments have improved substantially over the study period and may have decreased PCSM and mPCa rates; our reported risks may therefore be overestimates under contemporary patterns of care. Seventh, we did not have data on digital rectal examinations, which may provide important risk information if abnormal. Finally, because veterans have higher comorbidity burdens than the general population and unique military-related environmental exposures, our results should be cautiously extrapolated to other populations.^24^

## Conclusions

In this cohort study, we found that most men who receive health care through the VHA continue PSA screening after age 70 years despite low absolute 10-year PCSM risks. Baseline PSA values from age 65 to 69 years may be highly informative for adverse prostate cancer outcomes after age 70 years, with a PSA of less than 1 ng/mL associated with a very low risk of long-term PCSM and mPCa.

## Notes

Supplement 1.
eMethods.
**eFigure 1.** Patient Selection Flow Diagram **eFigure 2.** Calibration Plots for Prediction Models **eFigure 3.** Longitudinal Representation of PSA Screens for 50 Randomly Selected Patients **eFigure 4.** Cumulative Incidence of Clinically Significant Prostate Cancer **eFigure 5.** Cumulative Incidence of Prostate Cancer Treatment **eFigure 6.** Rates of All-Cause Mortality **eTable 1.** Top 20 Variables in the XGBoost Survival Model **eTable 2.** Characteristics of the Sample by Self-Reported Race **eTable 3.** Characteristics of the Sample by 10-Year Predicted PCSM Risk **eTable 4.** Characteristics of the Sample by 10-Year Predicted Overall Survival **eTable 5.** Results of Negative Binomial Regression for Intensity of PSA Screening During Follow-Up After Age 70

Supplement 2.
Data Sharing Statement
