# Error in Figure 1

**DOI:** 10.1001/jamanetworkopen.2025.8160

**Published:** 2025-03-27

In the Original Investigation titled “Prostate Cancer Mortality in Men Aged 70 Years Who Recently Underwent Prostate-Specific Antigen Screening,”^1^ published February 14, 2025, there were errors in the labels of panels D, E, and F of Figure 1. The labels should have indicated that incidence of prostate biopsy was shown rather than prostate cancer–specific mortality. This article has been corrected.^1^
