# Supplementary material for: Prostate Cancer Mortality in Men Aged 70 Years Who Recently Underwent Prostate-Specific Antigen Screening
Source: JAMA Netw Open. 2025 Feb 14;8(2):e2459766. doi: 10.1001/jamanetworkopen.2024.59766 (PMC11829239; doi:10.1001/jamanetworkopen.2024.59766)
Supplement: Supplement 1. — eMethods. eFigure 1. Patient Selection Flow Diagram eFigure 2. Calibration Plots for Prediction Models eFigure 3. Longitudinal Representation of PSA Screens for 50 Randomly Selected Patients eFigure 4. Cumulative Incidence of Clinically Significant Prostate Cancer eFigure 5. Cumulative Incidence of Prostate Cancer Treatment eFigure 6. Rates of All-Cause Mortality eTable 1. Top 20 Variables in the XGBoost Survival Model eTable 2. Characteristics of the Sample by Self-Reported Race eTable 3. Characteristics of the Sample by 10-Year Predicted PCSM Risk eTable 4. Characteristics of the Sample by 10-Year Predicted Overall Survival eTable 5. Results of Negative Binomial Regression for Intensity of PSA Screening During Follow-Up After Age 70 [file jamanetwopen-e2459766-s001.pdf]

Supplemental Online Content

Chung DH, Caverly TJ, Schipper MJ, et al. Prostate cancer mortality in men aged 70 who recently underwent prostate-specific antigen screening. *JAMA Netw Open*. 2025;8(2):e2459766. doi:10.1001/jamanetworkopen.2024.59766

eMethods

eFigure 1. Patient Selection Flow Diagram

eFigure 2. Calibration Plots for Prediction Models

eFigure 3. Longitudinal Representation of PSA Screens for 50 Randomly Selected Patients

eFigure 4. Cumulative Incidence of Clinically Significant Prostate Cancer

eFigure 5. Cumulative Incidence of Prostate Cancer Treatment

eFigure 6. Rates of All-Cause Mortality

eTable 1. Top 20 Variables in the XGBoost Survival Model

eTable 2. Characteristics of the Sample by Self-Reported Race

eTable 3. Characteristics of the Sample by 10-Year Predicted PCSM Risk

eTable 4. Characteristics of the Sample by 10-Year Predicted Overall Survival

eTable 5. Results of Negative Binomial Regression for Intensity of PSA Screening During Follow-Up After Age 70

This supplemental material has been provided by the authors to give readers additional information about their work.

© 2025 Chung DH et al. *JAMA Network Open*.

eMethods

Competing mortality risk model

Any potential benefit of PSA screening after the age of 70 is likely to be limited among patients with poor life expectancy.[16,17] To determine competing mortality risk, we internally developed a machine learning model to stratify patients on their 10-year overall survival probability using VHA structured electronic medical record data in the year prior to the index date. Data included diagnosis and procedure codes, laboratory values, inpatient and outpatient medications, inpatient hospitalizations, and outpatient visits categorized by clinic type, as well as summary variables for each numeric variable (mean, minimum, maximum, most recent) or binary feature (count). We excluded binary variables with a prevalence less than 5% and numeric variables with missingness greater than 75%; this resulted in a final set of 1,266 variables. For further dimensionality reduction, we then trained an XGBoost-AFT survival model[18] on all-cause mortality and selected the top 100 most important variables using the gain metric. Using the narrowed variable list we then trained an XGBoost-Weibull parametric survival model,[19] which we found resulted in improved model calibration compared to XGBoost-AFT. All models were trained on a random 30% sample of the study cohort, and final performance metrics including concordance index and Brier score were calculated on the remaining held-out 70%.

The top predictors are described in **eTable 1** and calibration plots of predicted vs. observed 10-year survival are presented in **eFigure 2**. Of note, we would caution against over-interpretation of the selected variables in eTable 1, as this represents only one metric of variable importance in a highly complex, non-linear model. Many of the selected variables have a straightforward clinical explanation as markers of underlying poor health (e.g. vital sign abnormalities, COPD diagnosis, furosemide prescriptions); others may be markers of increased health utilization (any patient encounter in the prior year) or preventative care-seeking behavior (influenza vaccination, strep pneumoniae vaccination).

© 2025 Chung DH et al. *JAMA Network Open.*

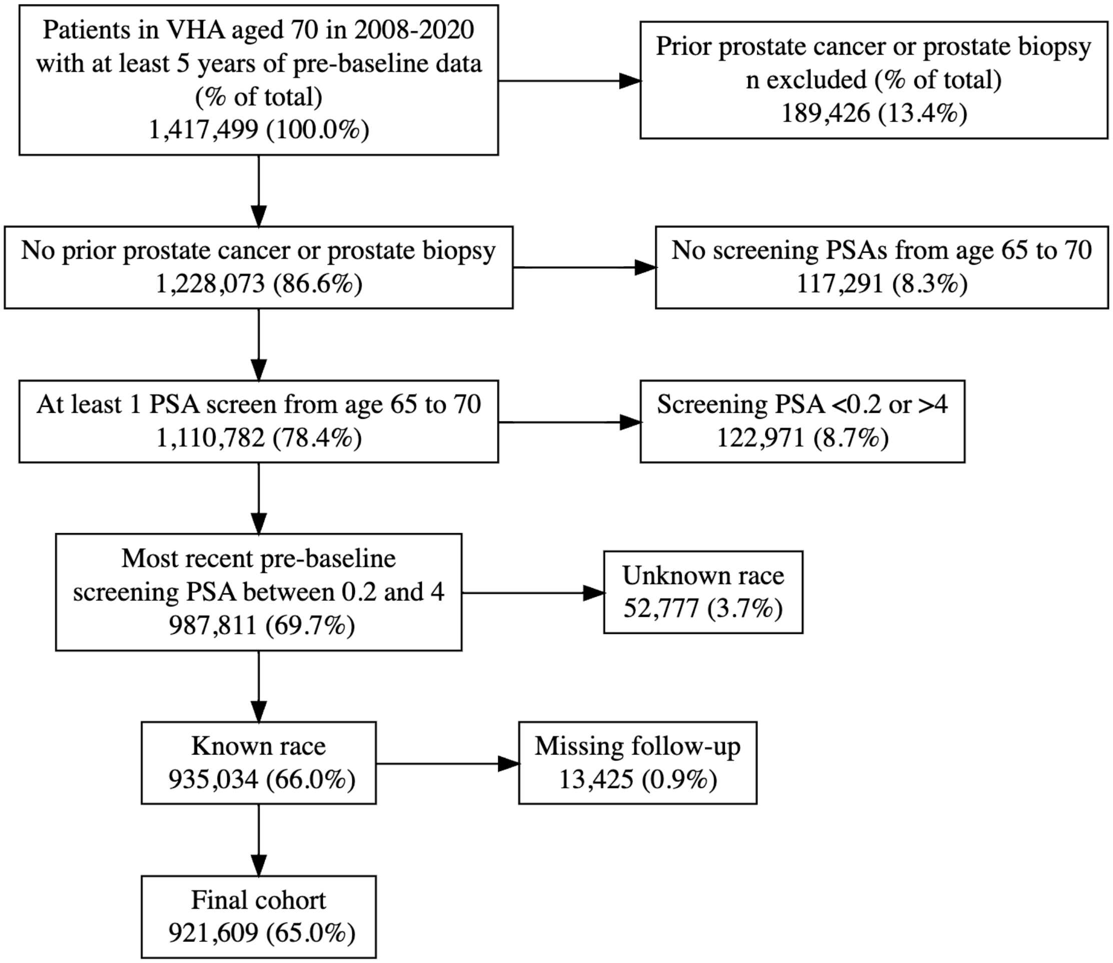

eFigure 1. Patient selection flow diagram.

© 2025 Chung DH et al. *JAMA Network Open*.

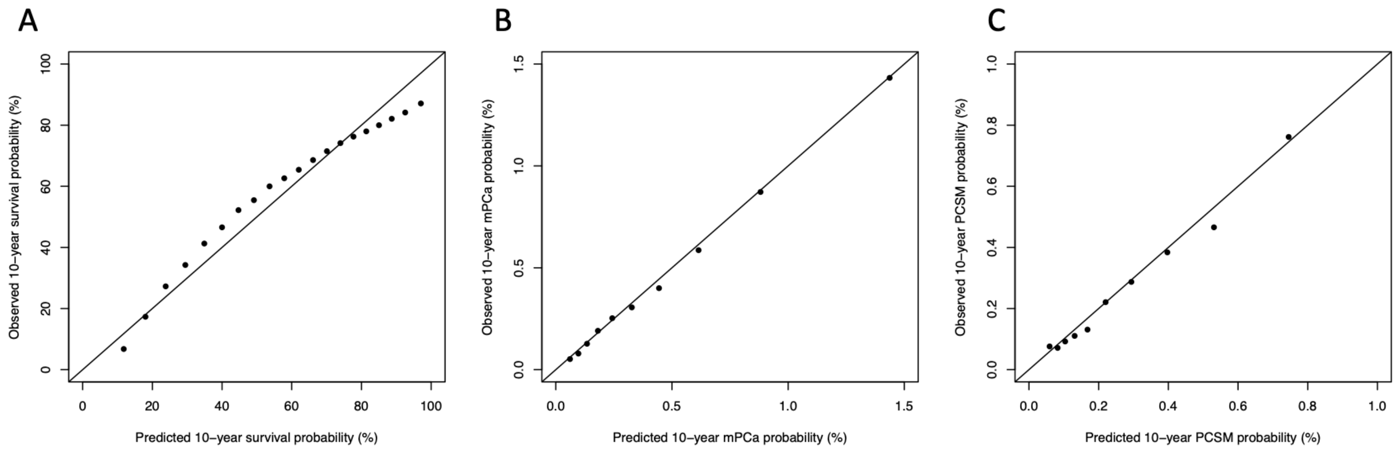

eFigure 2. Calibration plots for prediction models. (**A**) Predicted (y-axis) vs. observed (x-axis) 10-year overall survival probability, by ventile of predicted survival. This model provided slightly pessimistic survival predictions among patients with intermediate predicted survival and slightly optimistic predictions among patients with excellent predicted survival. (**B**) Predicted vs. observed 10-year mPCa probability by ventile of predicted risk. (**C**) Predicted vs. observed 10-year PCSM probability by ventile of predicted risk. Abbreviations: mPCa: metastatic prostate cancer; PCSM: prostate cancer-specific mortality.

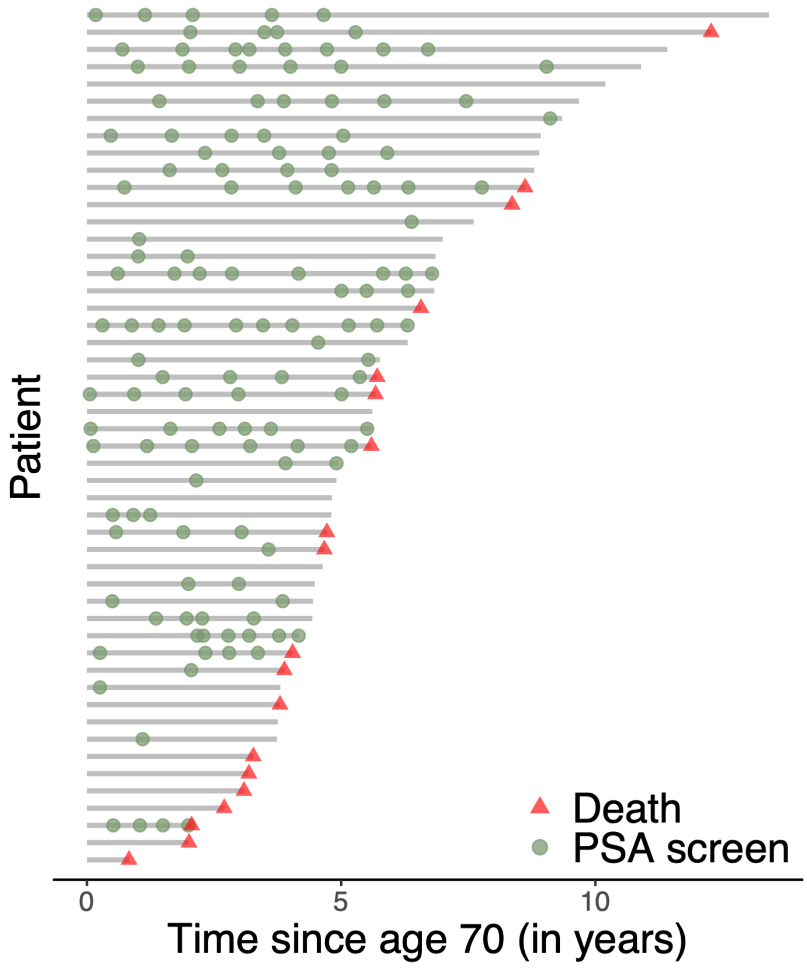

eFigure 3. Longitudinal representation of PSA screens for 50 randomly selected patients. Green circles represent PSA screens; red triangles represent all-cause mortality events.

© 2025 Chung DH et al. *JAMA Network Open*.

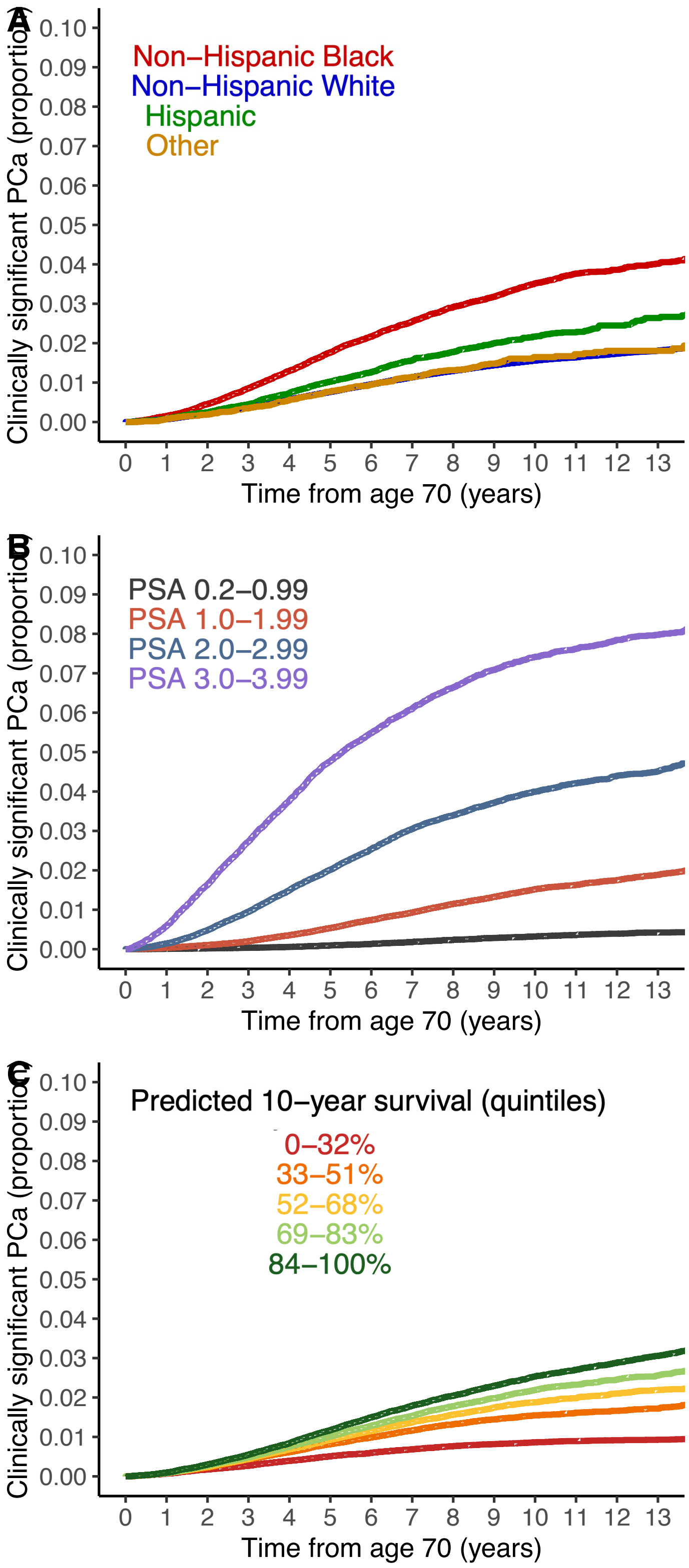

eFigure 4. Cumulative incidence of clinically significant prostate cancer by (**A**) race, (**B**) baseline PSA, and (**C**) predicted 10-year overall survival. Clinically significant prostate cancer is defined as any prostate cancer with Gleason score 3+4 or higher.

© 2025 Chung DH et al. *JAMA Network Open*.

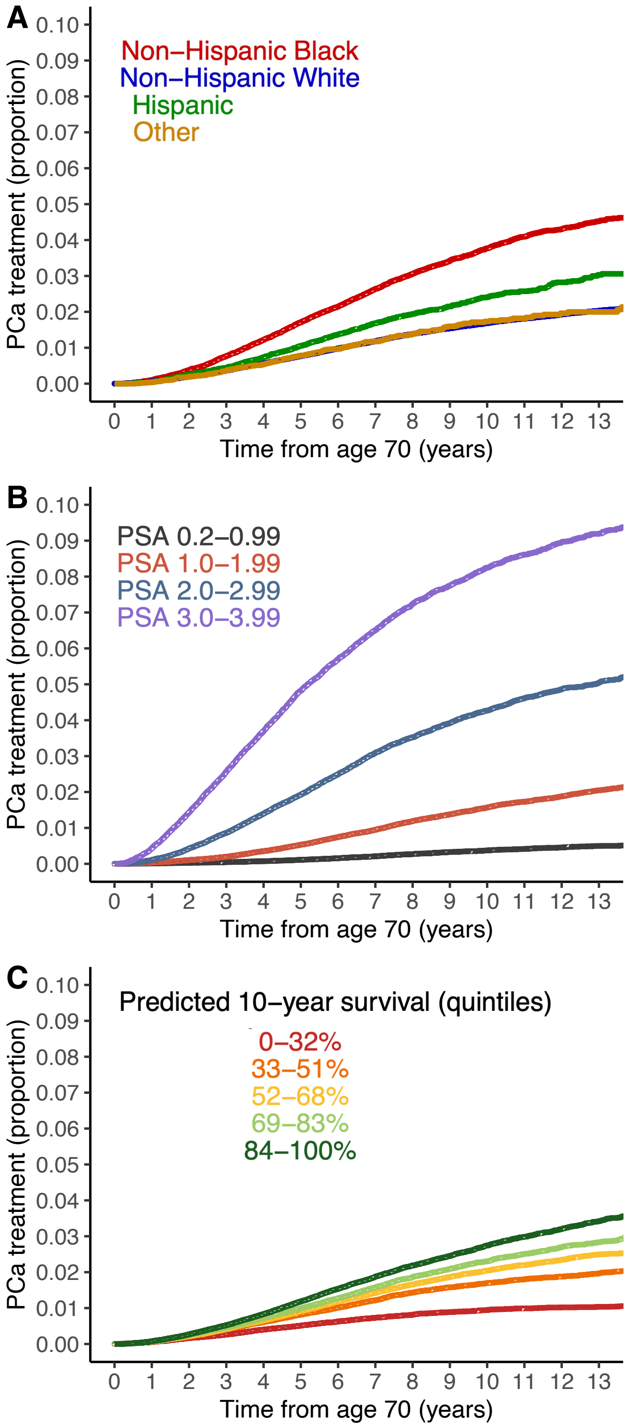

eFigure 5. Cumulative incidence of prostate cancer treatment by (**A**) race, (**B**) baseline PSA, and (**C**) predicted 10-year overall survival. Prostate cancer treatment includes radiotherapy for prostate cancer, radical prostatectomy, and hormonal therapy.

© 2025 Chung DH et al. *JAMA Network Open*.

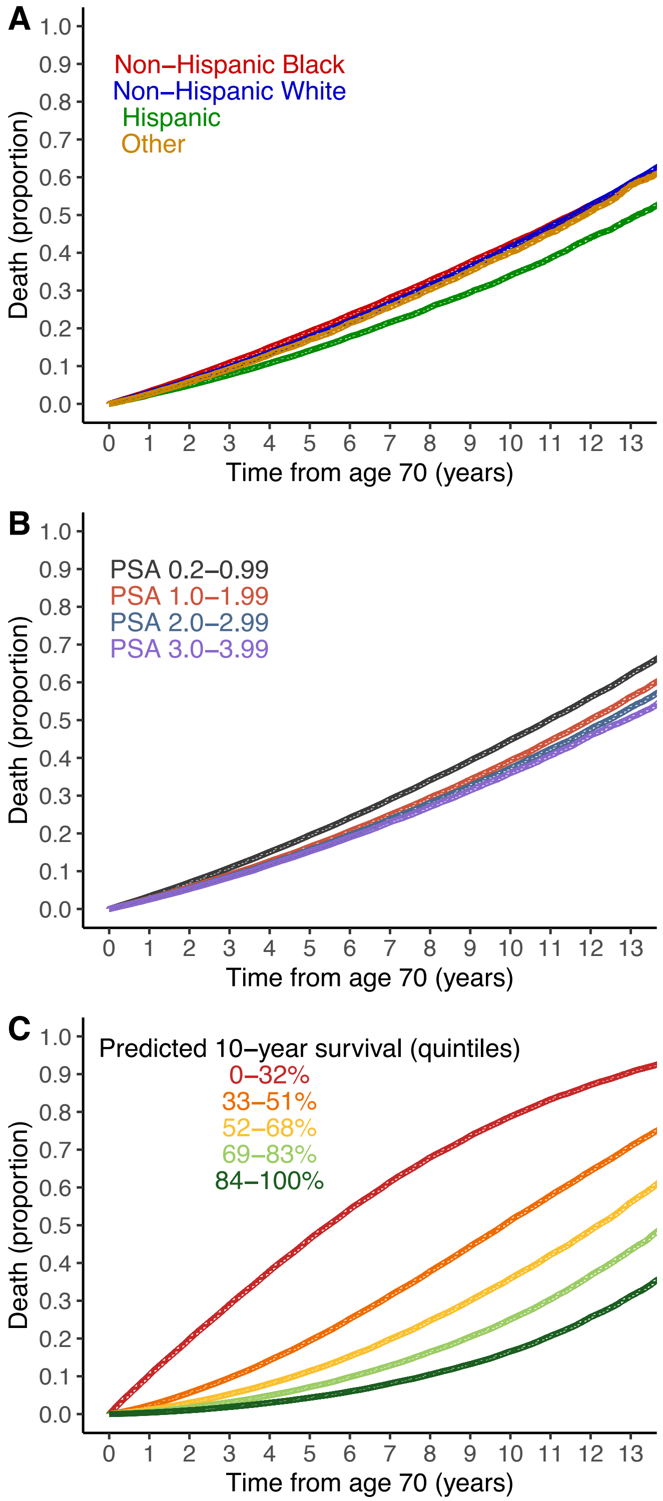

eFigure 6. Cumulative rates of all-cause mortality by (**A**) race, (**B**) baseline PSA, and (**C**) predicted 10-year overall survival.

© 2025 Chung DH et al. *JAMA Network Open.*

eTable 1. Top 20 variables in the XGBoost survival model.

Variable	Gain[1]	Code	Code type
Measurement of immature granulocytes/100 leukocytes in blood (binary)	3172	38518-7	LOINC
Any patient encounter (binary)	793	4203722	OMOP
Influenza vaccination, preservative free (binary)	486	40213154	OMOP
Albumin in serum or plasma (minimum)	463	1751-7	LOINC
Sildenafil prescription (binary)	409	--	VA formulary
Chronic obstructive lung disease (binary)	329	255573	OMOP
Influenza vaccination, any (binary)	222	40213153	OMOP
Erythrocyte distribution width (maximum)	213	788-0	LOINC
Streptococcus pneumoniae vaccination (binary)	197	40173508	OMOP
Hemoglobin in blood (minimum)	183	718-7	LOINC
Erythrocyte count in blood (minimum)	179	789-8	LOINC
Furosemide prescription (binary)	165	--	VA formulary
Erythrocyte count in blood (most recent)	125	789-8	LOINC
Hemoglobin in blood (most recent)	120	718-7	LOINC
Chloride in serum or plasma (minimum)	113	2075-0	LOINC
Tiotropium prescription (binary)	112	--	VA formulary
Respiratory rate (maximum)	107	--	--
Heart rate (maximum)	104	--	--
Alkaline phosphatase in serum or plasma (maximum)	102	6768-6	LOINC
Requires influenza vaccination (binary)	102	44784283	OMOP

[1]Gain is a measure of the contribution of each feature in reducing prediction error.
Baseline features are captured in the year prior to the index date.
Abbreviations: LOINC: Logical Observation Identifiers Names and Coces; OMOP: Observational Medical Outcomes Partnership; VA: Veterans Affairs.

© 2025 Chung DH et al. *JAMA Network Open*.

eTable 2. Characteristics of the sample by self-reported race.

Characteristic	Hispanic<br>N = 44,719[1]	Non-Hispanic White<br>N = 753,344[1]	Non-Hispanic Black<br>N = 102,692[1]	Other<br>N = 20,854[1]
**Most recent PSA, group (ng/dL)**				
0.2-0.99	19,783 (44%)	343,362 (46%)	42,858 (42%)	9,441 (45%)
1-1.99	14,573 (33%)	241,619 (32%)	33,619 (33%)	6,679 (32%)
2-2.99	6,717 (15%)	110,636 (15%)	16,809 (16%)	3,112 (15%)
3-3.99	3,646 (8.2%)	57,727 (7.7%)	9,406 (9.2%)	1,622 (7.8%)
**Most recent PSA (ng/dL)**	1.11 (0.64, 1.90)	1.09 (0.61, 1.86)	1.18 (0.68, 2.00)	1.10 (0.62, 1.89)
**Number of PSAs in prior 5 years**	4 (3, 6)	4 (3, 5)	4 (3, 5)	4 (3, 5)
**Year of index date**				
2008-2010	4,024 (9.0%)	83,516 (11%)	8,456 (8.2%)	2,102 (10%)
2011-2013	6,436 (14%)	120,704 (16%)	15,456 (15%)	3,182 (15%)
2014-2016	11,497 (26%)	199,693 (27%)	26,486 (26%)	5,620 (27%)
2017-2020	22,762 (51%)	349,431 (46%)	52,294 (51%)	9,950 (48%)
**Geographic region**				
Continental	12,569 (28%)	122,343 (16%)	21,618 (21%)	4,153 (20%)
Midwest	2,292 (5.1%)	200,362 (27%)	16,741 (16%)	3,176 (15%)
North Atlantic	2,992 (6.7%)	174,690 (23%)	27,177 (26%)	2,689 (13%)
Pacific	13,225 (30%)	112,515 (15%)	9,282 (9.0%)	8,260 (40%)
Southeast	13,641 (31%)	143,434 (19%)	27,874 (27%)	2,576 (12%)
**Charlson Comorbidity Index**	1 (0, 3)	1 (0, 3)	2 (1, 3)	1 (0, 3)
**Area Deprivation Index**				
1-20	5,919 (13%)	62,247 (8.4%)	8,279 (8.2%)	4,309 (21%)
21-40	7,121 (16%)	127,825 (17%)	13,321 (13%)	3,785 (18%)
41-60	7,694 (17%)	178,414 (24%)	18,275 (18%)	3,872 (19%)
61-80	7,716 (17%)	201,374 (27%)	23,357 (23%)	4,188 (20%)
81-100	15,691 (36%)	169,439 (23%)	37,133 (37%)	4,329 (21%)
**RUCA classification**				
Urban	39,786 (89%)	527,032 (70%)	90,235 (88%)	15,518 (76%)
Large rural city/town	2,846 (6.4%)	111,124 (15%)	7,295 (7.1%)	2,569 (13%)
Small rural town	1,204 (2.7%)	61,405 (8.2%)	3,538 (3.5%)	1,231 (6.0%)
Isolated small rural town	694 (1.6%)	51,136 (6.8%)	1,205 (1.2%)	1,213 (5.9%)
**Distance from VHA facility (miles)**				
0-25 miles	34,294 (79%)	465,731 (63%)	78,113 (77%)	13,472 (66%)
26-50 miles	4,855 (11%)	148,621 (20%)	12,335 (12%)	3,346 (16%)
51-75 miles	1,695 (3.9%)	62,545 (8.4%)	5,548 (5.5%)	1,329 (6.5%)
>75 miles	2,676 (6.1%)	64,688 (8.7%)	5,425 (5.3%)	2,244 (11%)
**VA Frailty Index**	0.16 (0.10, 0.26)	0.16 (0.10, 0.26)	0.16 (0.10, 0.26)	0.16 (0.10, 0.26)
**History of BPH**	15,699 (35%)	261,519 (35%)	38,330 (37%)	7,044 (34%)
**History of prostatitis**	2,100 (4.7%)	28,691 (3.8%)	4,563 (4.4%)	776 (3.7%)
**5-ARI use at baseline**	3,436 (7.7%)	47,508 (6.3%)	7,889 (7.7%)	1,402 (6.7%)
**Alpha-1 antagonist use at baseline**	10,456 (23%)	159,855 (21%)	26,738 (26%)	4,541 (22%)
**PDE-5 inhibitor use at baseline**	10,885 (24%)	126,951 (17%)	38,682 (38%)	4,292 (21%)
**ED visit in prior year**	8,771 (20%)	108,352 (14%)	24,717 (24%)	2,970 (14%)
**PCP visit in prior year**	40,231 (90%)	652,659 (87%)	91,074 (89%)	18,113 (87%)
**Urologist visit in prior year**	3,700 (8.3%)	52,262 (6.9%)	9,988 (9.7%)	1,325 (6.4%)

[1]n (%); Median (Q1, Q3)

Abbreviations: PSA: prostate specific antigen; VA: Veterans Affairs: BPH: benign prostatic hypertrophy; 5-ARI: 5-alpha reductase inhibitors; PDE-5: phosphodiesterase-5 inhibitors; mPCa: metastatic prostate cancer.

eTable 3. Characteristics of the sample by 10-year predicted PCSM risk.

Characteristic	**[0.0259,0.0925)**, N = 184,322[1]	**[0.0925,0.1475)**, N = 184,334[1]	**[0.1475,0.2524)**, N = 184,310[1]	**[0.2524,0.4591)**, N = 184,362[1]	**[0.4591,1.1975]**, N = 184,281[1]
**Self-reported race**					
Hispanic	13,174 (7.1%)	8,365 (4.5%)	8,473 (4.6%)	9,237 (5.0%)	5,470 (3.0%)
Non-Hispanic White	154,275 (84%)	152,603 (83%)	150,705 (82%)	149,534 (81%)	146,227 (79%)
Non-Hispanic Black	12,198 (6.6%)	19,182 (10%)	21,061 (11%)	21,378 (12%)	28,873 (16%)
Other	4,675 (2.5%)	4,184 (2.3%)	4,071 (2.2%)	4,213 (2.3%)	3,711 (2.0%)
**Most recent PSA, group (ng/dL)**					
0.2-0.99	184,269 (100%)	168,331 (91%)	62,833 (34%)	11 (<0.1%)	0 (0%)
1-1.99	53 (<0.1%)	16,003 (8.7%)	121,354 (66%)	146,003 (79%)	13,077 (7.1%)
2-2.99	0 (0%)	0 (0%)	123 (<0.1%)	36,667 (20%)	100,484 (55%)
3-3.99	0 (0%)	0 (0%)	0 (0%)	1,681 (0.9%)	70,720 (38%)
**Most recent PSA (ng/dL)**	0.40 (0.30, 0.50)	0.70 (0.59, 0.81)	1.09 (0.93, 1.22)	1.67 (1.44, 1.90)	2.76 (2.30, 3.28)
**Number of PSAs in prior 5 years**	4 (3, 5)	4 (3, 5)	4 (3, 5)	4 (3, 5)	4 (3, 6)
**Year of index date**					
2008-2010	19,852 (11%)	19,688 (11%)	19,844 (11%)	19,549 (11%)	19,165 (10%)
2011-2013	29,785 (16%)	29,422 (16%)	29,315 (16%)	29,176 (16%)	28,080 (15%)
2014-2016	49,395 (27%)	48,823 (26%)	48,415 (26%)	48,884 (27%)	47,779 (26%)
2017-2020	85,290 (46%)	86,401 (47%)	86,736 (47%)	86,753 (47%)	89,257 (48%)
**Geographic region**					
Continental	33,515 (18%)	32,334 (18%)	31,807 (17%)	31,616 (17%)	31,411 (17%)
Midwest	44,378 (24%)	44,871 (24%)	44,526 (24%)	44,678 (24%)	44,118 (24%)
North Atlantic	40,719 (22%)	41,356 (22%)	41,598 (23%)	41,507 (23%)	42,368 (23%)
Pacific	28,747 (16%)	28,244 (15%)	28,484 (15%)	29,061 (16%)	28,746 (16%)
Southeast	36,963 (20%)	37,529 (20%)	37,895 (21%)	37,500 (20%)	37,638 (20%)
**Area Deprivation Index**					
1-20	15,731 (8.7%)	15,866 (8.8%)	16,167 (8.9%)	16,637 (9.2%)	16,353 (9.0%)
21-40	29,853 (17%)	30,151 (17%)	30,461 (17%)	30,717 (17%)	30,870 (17%)
41-60	41,167 (23%)	41,727 (23%)	41,976 (23%)	42,039 (23%)	41,346 (23%)
61-80	47,929 (27%)	47,861 (26%)	47,115 (26%)	46,997 (26%)	46,733 (26%)
81-100	46,128 (26%)	45,383 (25%)	45,170 (25%)	44,395 (25%)	45,516 (25%)
**RUCA classification**					
Urban	133,694 (73%)	133,895 (73%)	134,570 (73%)	134,937 (73%)	135,475 (74%)
Large rural city/town	25,191 (14%)	25,054 (14%)	24,916 (14%)	24,510 (13%)	24,163 (13%)
Small rural town	13,760 (7.5%)	13,696 (7.5%)	13,364 (7.3%)	13,299 (7.2%)	13,259 (7.2%)
Isolated small rural town	10,951 (6.0%)	10,994 (6.0%)	10,756 (5.9%)	10,913 (5.9%)	10,634 (5.8%)
**Distance from VHA facility (miles)**					
0-25 miles	116,149 (64%)	117,731 (65%)	118,646 (65%)	119,213 (66%)	119,871 (66%)
26-50 miles	35,060 (19%)	34,050 (19%)	33,574 (19%)	33,549 (18%)	32,924 (18%)
51-75 miles	14,517 (8.0%)	14,482 (8.0%)	14,402 (7.9%)	13,777 (7.6%)	13,939 (7.7%)
>75 miles	15,528 (8.6%)	15,086 (8.3%)	14,801 (8.2%)	14,931 (8.2%)	14,687 (8.1%)
**Charlson Comorbidity Index**	2 (0, 4)	1 (0, 3)	1 (0, 3)	1 (0, 3)	1 (0, 3)
**VA Frailty Index**	0.16 (0.10, 0.29)	0.16 (0.10, 0.26)	0.16 (0.10, 0.26)	0.16 (0.10, 0.26)	0.16 (0.10, 0.23)
**History of BPH**	59,390 (32%)	57,982 (31%)	60,963 (33%)	67,537 (37%)	76,720 (42%)
**History of prostatitis**	6,339 (3.4%)	6,309 (3.4%)	6,849 (3.7%)	7,715 (4.2%)	8,918 (4.8%)
**5-ARI use at baseline**	11,137 (6.0%)	9,276 (5.0%)	10,272 (5.6%)	12,430 (6.7%)	17,120 (9.3%)
**Alpha-1 antagonist use at baseline**	36,880 (20%)	35,897 (19%)	37,804 (21%)	42,178 (23%)	48,831 (26%)
**PDE-5 inhibitor use at baseline**	32,769 (18%)	35,530 (19%)	36,625 (20%)	37,872 (21%)	38,014 (21%)
**ED visit in prior year**	32,281 (18%)	28,443 (15%)	28,033 (15%)	28,117 (15%)	27,936 (15%)
**PCP visit in prior year**	163,097 (88%)	160,619 (87%)	159,872 (87%)	159,744 (87%)	158,745 (86%)

© 2025 Chung DH et al. *JAMA Network Open*.

Characteristic	**[0.0259,0.0925)**, N = 184,322[1]	**[0.0925,0.1475)**, N = 184,334[1]	**[0.1475,0.2524)**, N = 184,310[1]	**[0.2524,0.4591)**, N = 184,362[1]	**[0.4591,1.1975]**, N = 184,281[1]
**Urologist visit in prior year**	12,174 (6.6%)	11,856 (6.4%)	12,203 (6.6%)	13,694 (7.4%)	17,348 (9.4%)

[1]n (%); Median (Q1, Q3)
Abbreviations: PSA: prostate specific antigen; VA: Veterans Affairs: BPH: benign prostatic hypertrophy; 5-ARI: 5-alpha reductase inhibitors; PDE-5: phosphodiesterase-5 inhibitors; mPCa: metastatic prostate cancer.

© 2025 Chung DH et al. *JAMA Network Open*.

eTable 4. Characteristics of the sample by 10-year predicted overall survival.

Characteristic	[7.06, 32.2) N = 184,323[1]	[32.21, 51.4) N = 184,322[1]	[51.44, 68.1) N = 184,321[1]	[68.13, 83.2) N = 184,323[1]	[83.22,100.0] N = 184,320[1]
**Self-reported race**					
Hispanic	7,410 (4.0%)	8,530 (4.6%)	9,285 (5.0%)	9,825 (5.3%)	9,669 (5.2%)
Non-Hispanic White	148,281 (80%)	148,648 (81%)	149,677 (81%)	151,675 (82%)	155,063 (84%)
Non-Hispanic Black	24,626 (13%)	22,855 (12%)	21,086 (11%)	18,581 (10%)	15,544 (8.4%)
Other	4,006 (2.2%)	4,289 (2.3%)	4,273 (2.3%)	4,242 (2.3%)	4,044 (2.2%)
**Most recent PSA, group (ng/dL)**					
0.2-0.99	95,051 (52%)	87,948 (48%)	83,437 (45%)	78,071 (42%)	70,937 (38%)
1-1.99	54,173 (29%)	57,793 (31%)	59,493 (32%)	61,415 (33%)	63,616 (35%)
2-2.99	23,114 (13%)	25,396 (14%)	27,167 (15%)	29,214 (16%)	32,383 (18%)
3-3.99	11,985 (6.5%)	13,185 (7.2%)	14,224 (7.7%)	15,623 (8.5%)	17,384 (9.4%)
**Most recent PSA (ng/dL)**	0.95 (0.54, 1.70)	1.03 (0.60, 1.80)	1.10 (0.62, 1.87)	1.15 (0.67, 1.96)	1.24 (0.72, 2.08)
**Number of PSAs in prior 5 years**	4 (3, 5)	4 (3, 5)	4 (3, 5)	4 (3, 6)	5 (4, 6)
**Year of index date**					
2008-2010	21,012 (11%)	19,987 (11%)	20,102 (11%)	19,250 (10%)	17,747 (9.6%)
2011-2013	30,216 (16%)	28,203 (15%)	28,230 (15%)	27,880 (15%)	31,249 (17%)
2014-2016	46,924 (25%)	46,048 (25%)	45,871 (25%)	47,102 (26%)	57,351 (31%)
2017-2020	86,171 (47%)	90,084 (49%)	90,118 (49%)	90,091 (49%)	77,973 (42%)
**Geographic region**					
Continental	34,145 (19%)	32,776 (18%)	32,025 (17%)	31,035 (17%)	30,702 (17%)
Midwest	43,875 (24%)	43,398 (24%)	43,490 (24%)	44,670 (24%)	47,138 (26%)
North Atlantic	41,409 (22%)	40,931 (22%)	41,157 (22%)	41,390 (22%)	42,661 (23%)
Pacific	27,261 (15%)	29,248 (16%)	29,532 (16%)	29,108 (16%)	28,133 (15%)
Southeast	37,633 (20%)	37,969 (21%)	38,117 (21%)	38,120 (21%)	35,686 (19%)
**Area Deprivation Index**					
1-20	13,013 (7.2%)	14,733 (8.1%)	16,001 (8.8%)	17,729 (9.8%)	19,278 (11%)
21-40	26,219 (15%)	28,717 (16%)	30,763 (17%)	32,099 (18%)	34,254 (19%)
41-60	39,259 (22%)	40,791 (23%)	41,958 (23%)	42,508 (23%)	43,739 (24%)
61-80	49,020 (27%)	48,255 (27%)	47,063 (26%)	46,566 (26%)	45,731 (25%)
81-100	53,015 (29%)	48,457 (27%)	45,218 (25%)	42,081 (23%)	37,821 (21%)
**RUCA classification**					
Urban	135,793 (74%)	135,142 (74%)	134,844 (73%)	133,994 (73%)	132,798 (72%)
Large rural city/town	24,891 (14%)	24,906 (14%)	24,576 (13%)	24,644 (13%)	24,817 (14%)
Small rural town	13,125 (7.1%)	13,247 (7.2%)	13,466 (7.3%)	13,757 (7.5%)	13,783 (7.5%)
Isolated small rural town	9,957 (5.4%)	10,323 (5.6%)	10,697 (5.8%)	11,122 (6.1%)	12,149 (6.6%)
**Distance from VHA facility (miles)**					
0-25 miles	114,627 (63%)	117,941 (65%)	119,215 (66%)	119,531 (66%)	120,296 (67%)
26-50 miles	34,814 (19%)	33,786 (19%)	33,606 (19%)	33,667 (19%)	33,284 (18%)
51-75 miles	15,922 (8.7%)	14,555 (8.0%)	13,709 (7.6%)	13,685 (7.6%)	13,246 (7.3%)
>75 miles	16,711 (9.2%)	15,288 (8.4%)	14,747 (8.1%)	14,253 (7.9%)	14,034 (7.8%)
**Charlson Comorbidity Index**	3 (2, 6)	2 (1, 3)	1 (0, 3)	1 (0, 2)	0 (0, 1)
**VA Frailty Index**	0.26 (0.16, 0.39)	0.16 (0.10, 0.29)	0.16 (0.10, 0.23)	0.13 (0.06, 0.19)	0.10 (0.06, 0.19)

© 2025 Chung DH et al. *JAMA Network Open.*

Characteristic	[7.06, 32.2)<br>N = 184,323[1]	[32.21, 51.4)<br>N = 184,322[1]	[51.44, 68.1)<br>N = 184,321[1]	[68.13, 83.2)<br>N = 184,323[1]	[83.22,100.0]<br>N = 184,320[1]
**History of BPH**	68,115 (37%)	62,591 (34%)	62,326 (34%)	63,243 (34%)	66,317 (36%)
**History of prostatitis**	6,610 (3.6%)	6,960 (3.8%)	7,254 (3.9%)	7,463 (4.0%)	7,843 (4.3%)
**5-ARI use at baseline**	15,466 (8.4%)	12,407 (6.7%)	11,291 (6.1%)	10,752 (5.8%)	10,319 (5.6%)
**Alpha-1 antagonist use at baseline**	50,120 (27%)	41,701 (23%)	38,286 (21%)	36,408 (20%)	35,075 (19%)
**PDE-5 inhibitor use at baseline**	23,630 (13%)	29,060 (16%)	34,882 (19%)	40,732 (22%)	52,506 (28%)
**ED visit in prior year**	56,035 (30%)	30,500 (17%)	23,657 (13%)	19,308 (10%)	15,310 (8.3%)
**PCP visit in prior year**	164,779 (89%)	155,098 (84%)	159,082 (86%)	160,634 (87%)	162,484 (88%)
**Urologist visit in prior year**	18,371 (10.0%)	13,862 (7.5%)	12,401 (6.7%)	11,778 (6.4%)	10,863 (5.9%)

[1]n (%); Median (Q1, Q3)

Abbreviations: PSA: prostate specific antigen; VA: Veterans Affairs: BPH: benign prostatic hypertrophy; 5-ARI: 5-alpha reductase inhibitors; PDE-5: phosphodiesterase-5 inhibitors; mPCa: metastatic prostate cancer.

eTable 5. Results of negative binomial regression for intensity of PSA screening during follow-up after age 70.

Characteristic	IRR[1]	95% CI[1]	p-value
**Most recent PSA, group (ng/mL)**			
0.2-0.99	—	—	
1-1.99	1.04	1.03, 1.04	<0.001
2-2.99	1.14	1.13, 1.14	<0.001
3-3.99	1.27	1.26, 1.28	<0.001
**Self-reported race**			
Hispanic	1.10	1.09, 1.11	<0.001
Non-Hispanic White	—	—	
Non-Hispanic Black	0.98	0.98, 0.99	<0.001
Other	0.97	0.96, 0.98	<0.001
**Predicted 10-year survival, quintile**			
1 (sickest)	—	—	
2	1.02	1.02, 1.03	<0.001
3	1.03	1.02, 1.03	<0.001
4	1.05	1.04, 1.05	<0.001
5 (healthiest)	1.07	1.06, 1.07	<0.001
**Year of index date**			
2008-2010	—	—	
2011-2013	0.93	0.93, 0.94	<0.001
2014-2016	1.00	0.99, 1.00	0.7
2017-2020	1.08	1.07, 1.08	<0.001
**Number of PSAs in prior 5 years**	1.12	1.12, 1.12	<0.001
**History of BPH**	1.00	1.00, 1.00	0.4
**History of prostatitis**	1.02	1.01, 1.03	<0.001
**5-ARI use at baseline**	0.96	0.96, 0.97	<0.001
**Alpha-1 antagonist use at baseline**	1.03	1.02, 1.03	<0.001
**PDE-5 inhibitor use at baseline**	1.03	1.03, 1.04	<0.001
**ED visit in prior year**	1.00	0.99, 1.00	0.4
**PCP visit in prior year**	1.14	1.13, 1.14	<0.001
**Urologist visit in prior year**	1.04	1.04, 1.05	<0.001

[1]IRR = Incidence Rate Ratio, CI = Confidence Interval

Abbreviations: PSA: prostate specific antigen; VA: Veterans Affairs: BPH: benign prostatic hypertrophy; 5-ARI: 5-alpha reductase inhibitors; PDE-5: phosphodiesterase-5 inhibitors; mPCa: metastatic prostate cancer.

© 2025 Chung DH et al. *JAMA Network Open*.
