# Supplementary material for: Prostate Cancer Mortality in Men Aged 70 Years Who Recently Underwent Prostate-Specific Antigen Screening
Source: JAMA Netw Open. 2025 Feb 14;8(2):e2459766. doi: 10.1001/jamanetworkopen.2024.59766 (PMC11829239; doi:10.1001/jamanetworkopen.2024.59766)
Supplement: Supplement 2. — Data Sharing Statement [file jamanetwopen-e2459766-s002.pdf]

Data Sharing Statement

Chung. Prostate Cancer Mortality in Men Aged 70 Years Who Recently Underwent Prostate-Specific Antigen Screening. *JAMA Netw Open*. Published February 14, 2025. doi:10.1001/jamanetworkopen.2024.59766

Data
Data available: No
